# An Isolated CNN Architecture for Classification of Finger-Tapping Tasks Using Initial Dip Images: A Functional Near-Infrared Spectroscopy Study

**DOI:** 10.3390/bioengineering10070810

**Published:** 2023-07-05

**Authors:** Muhammad Umair Ali, Amad Zafar, Karam Dad Kallu, M. Atif Yaqub, Haris Masood, Keum-Shik Hong, Muhammad Raheel Bhutta

**Affiliations:** 1 Department of Intelligent Mechatronics Engineering, Sejong University, Seoul 05006, Republic of Korea; umair@sejong.ac.kr; 2 Department of Robotics and Intelligent Machine Engineering (RIME), School of Mechanical and Manufacturing Engineering (SMME), National University of Sciences and Technology (NUST), H-12, Islamabad 44000, Pakistan; karamdad.kallu@smme.nust.edu.pk; 3 ICFO-Institut de Ciències Fotòniques the Barcelona Institute of Science and Technology, 08860 Castelldefels, Spain; atif.yaqub@icfo.eu; 4 Electrical Engineering Department, Wah Engineering College, University of Wah, Wah Cantt 47040, Pakistan; haris.masood@wecuw.edu.pk; 5 School of Mechanical Engineering, Pusan National University, 2 Busandaehak-ro, Geumjeong-gu, Busan 46241, Republic of Korea; kshong@pusan.ac.kr; 6 Institute for Future, School of Automation, Qingdao University, Qingdao 266071, China; 7 Department of Electrical and Computer Engineering, University of UTAH Asia Campus, Incheon 21985, Republic of Korea

**Keywords:** initial dip, neuronal firing, fNIRS, designed HRF, motor cortex, deep learning

## Abstract

This work investigates the classification of finger-tapping task images constructed for the initial dip duration of hemodynamics (HR) associated with the small brain area of the left motor cortex using functional near-infrared spectroscopy (fNIRS). Different layers (i.e., 16-layers, 19-layers, 22-layers, and 25-layers) of isolated convolutional neural network (CNN) designed from scratch are tested to classify the right-hand thumb and little finger-tapping tasks. Functional *t*-maps of finger-tapping tasks (thumb, little) were constructed for various durations (0.5 to 4 s with a uniform interval of 0.5 s) for the initial dip duration using a three gamma functions-based designed HR function. The results show that the 22-layered isolated CNN model yielded the highest classification accuracy of 89.2% with less complexity in classifying the functional *t*-maps of thumb and little fingers associated with the same small brain area using the initial dip. The results further demonstrated that the active brain area of the two tapping tasks from the same small brain area are highly different and well classified using functional *t*-maps of the initial dip (0.5 to 4 s) compared to functional *t*-maps generated for delayed HR (14 s). This study shows that the images constructed for initial dip duration can be helpful in the future for fNIRS-based diagnosis or cortical analysis of abnormal cerebral oxygen exchange in patients.

## 1. Introduction

Recently, functional near-infrared spectroscopy (fNIRS) has been shown to be a viable non-invasive imaging tool for studying brain activity in individuals of various ages [1,2]. The applications of fNIRS range from behavioral and cognitive neuroscience studies [3] to more innovative applications, including the investigation of cortical hemodynamic variations caused by driving [4], climbing [5], aircraft flying [6], decoding of sleep states including drowsiness [7,8], the administration of psychedelics, and interface with computers [9]. Furthermore, fNIRS has shown promising results when used in conjunction with various imaging modalities such as optical imaging [9], functional magnetic resonance imaging [10], electroencephalography [11,12,13], magnetoencephalography [14], or others [15,16,17].

fNIRS measures both absolute and relative concentration variations of oxyhemoglobin (HbO/∆HbO) and deoxyhemoglobin (HbR/∆HbR) using pairs of numerous near-infrared lights in the range of 650 to 1000 nm, which penetrate through the superficial cortical areas [18,19]. The brain’s neocortex is activated by stimuli, increasing blood flow, blood volume, HbO/∆HbO, and HbR/∆HbR in the regional neocortex [20]. The measured fNIRS signals (i.e., ∆HbO and ∆HbR) pass through three durations, including the initial dip (i.e., ∆HbO decreases and ∆HbR increases), the conventional hemodynamic response (HR) (i.e., ∆HbO increases and ∆HbR decreases), and the undershoot [9,21]. As the first increase (or reduction) in ∆HbR (or ∆HbO) results from an increase in metabolism, followed by an increase in cerebral blood flow, the early dip response is thought to be a faster and better spatial localizer of neuronal activity than the delayed conventional HR [22].

In fNIRS, the identification of neuronal activity in a specific brain area requires the calculation of a precise time series from the obtained ∆HbO and ∆HbR data known as canonical hemodynamic response function (cHRF), which may be derived from the *t*-statistics [23,24]. The cHRF can be modeled using two or three gamma functions. The desired HRF (dHRF) is then generated by convolving the cHRF with the designed experimental protocol [25]. Finally, the active channels are identified by fitting the dHRF on the experimental measured ∆HbO and ∆HbR signals. Using the results of *t*-statistics, the functional/spatial maps showing brain activity can be drawn [26]. With the advancement of deep learning in recent years, functional/spatial maps can now play an important role in categorizing or identifying brain areas associated with certain activities [27,28,29]. 

Deep learning has been effectively applied to fNIRS signals for classification in pre-processing/augmentation, brain–computer interface (BCI) applications, brain response analysis, and diagnosis [30]. In pre-processing/augmentation, Kim et al. [31] employed a U-Net as a convolutional neural network (CNN) architecture for de-noising the time series using the HRF with a minimum-square error of approximately 0.004–0.005. Furthermore, with reduced motion artifacts, their suggested approach correctly estimated the amplitude and shape of HRF. In the case of BCI, feature extraction is a tedious task. However, recently many studies utilized the benefit of automatic feature extraction characteristics of deep learning to eliminate the manual feature extraction process. Tanveer et al. [27] used color map images constructed at various intervals to classify drowsy and alert states using CNN. They achieved 99.3% average accuracy for drowsy/non-drowsy state detection. Another group achieved a high accuracy of 89% for classifying different tasks using deep neural network (DNN) architecture [32]. Similarly, the work proposed in [28] achieved a high classification accuracy of 99.63% for distinguishing between rest, mental arithmetic, and motor imagery tasks using recurrent CNN. Recently, Khalil et al. [33] classified different workload levels using CNN with an accuracy of 94.52%. For brain analysis using deep learning, researchers have employed CNN to investigate the brain’s responses due to stimuli, for example, like/dislike, high/low valence, male or female, discrete preference ratings, etc. [34,35]. 

Deep learning has also demonstrated its effectiveness in fNIRS-based diagnosis in patients [29,36,37,38]. In study [39], the activation *t*-maps and channel correlation maps were used to diagnose patients’ mild cognitive impairment (MCI). Three mental tasks, including the N-back tasks, Stroop task, and verbal fluency task, and 15 biomarkers were examined. They obtained 90.62% and 85.58% accuracy for activation *t*-maps and channel correlation maps, respectively. The same group in another study also utilized temporal-feature maps constructed at thirteen-time points to detect MCI in patients with achievable high accuracy [29]. For the N-back tasks, they achieved an accuracy of 98.61% using the slope map of ∆HbO for an interval of 20–60 s. Similarly, the authors in [40] classified the depressed and non-depressed states using pre-trained CNN models (i.e., ResNet 18, AlexNet) and a support vector machine. They utilized manual (i.e., time and frequency domain features) and channel correlation to train the models. They showed that ResNet 18, AlexNet, and support vector machine models achieved 76%, 90%, and 83% accuracy between depressed and non-depressed states. In another work [41], the authors utilized connectivity maps generated at various time instances from the resting state data with CNN to MCI detection in patients. Recently, Ho et al. [42] employed deep learning to categorize Alzheimer’s disease stages. All the studies mentioned above utilized various temporal window sizes on conventional HR to generate maps or time series for the same or different brain areas, which were input to deep learning architecture. So far, no study has investigated the effect of initial dip duration on functional maps using fNIRS.

In this study, an isolated CNN network with different layers is investigated for the classification of functional images generated for finger-tapping tasks using the initial duration (i.e., initial dip) of the HR; 16-, 19-, 22-, and 25-layer CNN architecture is designed from scratch. Then, the effectiveness of the designed isolated CNN architecture is evaluated using the right-hand thumb and little finger-tapping tasks using fNIRS signals generated from the left motor cortex. The functional maps for the right-hand thumb finger- (RHTF) and little finger- (RHLF) tapping are generated using *t*-statistic for the initial durations (i.e., 0.5 to 4 s with a uniform interval of 0.5 s) and the HR (14 s), which were further classified using the designed isolated CNN models. Finally, this work shows how functional *t*-maps constructed for the initial dip may be used to categorize several tasks performed by the same local brain region.

The paper is divided into the following sections. Section 2 discusses the fNIRS data acquisition and pre-processing, image construction, isolated CNN models, and proposed framework. The results of the proposed framework are presented in Section 3. Finally, Section 4 and Section 5 discuss and conclude the findings of this study.

## 2. Materials and Methods

**Figure 1:**
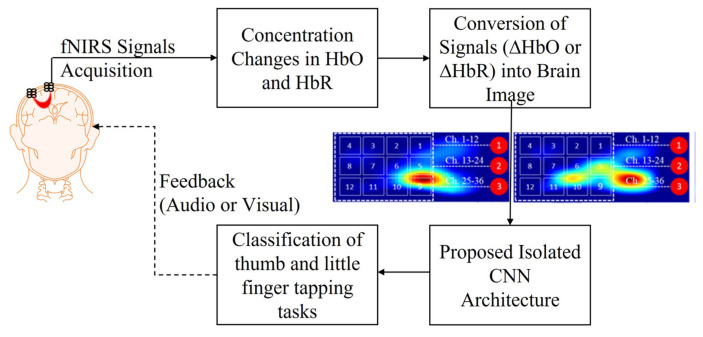
Proposed isolated CNN architecture framework.

Figure 1 shows the proposed isolated CNN architecture framework for the classification of RHTF- and RHLF-tapping tasks from the same brain area (i.e., left motor cortex).

### 2.1. Experimental Data and Pre-Processing

The proposed isolated CNN framework was validated using previously published experimental data of finger tapping (i.e., RHTF and RHLF) from eleven (11) volunteers (age: 28.5 ± 2.5 years, gender: male) [43]. None had a history of neurological or visual disorders; all were in good health with normal or corrected-to-normal vision. The fNIRS data of RHTF- and RHLF-tapping tasks were captured from the left motor cortex at a 9.19 Hz sampling rate using frequency domain ISS Imagent, ISS Inc. (Champaign IL, USA), equipment using near-infrared light of wavelengths 690 nm and 830 nm. The raw intensity data were acquired using ISS Imagent data collection and analysis software (ISS Boxy). The ISS-Boxy software was then used to convert the intensity data to ΔHbO and ΔHbR using the modified Beer–Lambert law. In a dense-emitter-detector arrangement, thirty-six channels using three detectors and twelve emitters were placed at the C3 area to examine the left motor cortex. The experiment took place in a dark, quiet room. The experimental protocol entails two sessions of tapping activities (i.e., one for RHTF-tapping and the other for RHLF-tapping) with 60 s of pre-rest and 10 s of post-rest. The six trials each last 30 s and make up a session. A 10 s tapping task is followed by a rest duration of 20 s in each trial. The individuals were asked to tap their RHTF and RHLF as quickly as they could without regard for the number of taps throughout the task time. A dark screen was displayed throughout the rest interval. The volunteers were also told to keep their eyes open during the experiment. To reduce physiological noises from respiration, heartbeat, and low-frequency drift, the measured data were pre-processed using a 4th-order band-pass filter having 0.01 and 0.15 Hz cutoff frequencies [44]. Further details on the optode configuration, paradigm protocol, and pre-processing can be found in [43,44].

### 2.2. Image Construction

The most critical step in analyzing fNIRS data is estimating cortical activity and localizing it. By adjusting the recorded HR to the predetermined dHRF, cortical activity may be determined [45], and its presence may be inferred from the associated channels’ *t*-values. A higher *t*-value specifies that the measured channel signal (i.e., ∆HbO) and dHRF have a strong correlation. In this paper, dHRF was generated by convolving cHRF made of three gamma functions with the experimental protocol (i.e., 10 s for RHTF or RHLF tasks and 20 s for the rest duration). The use of cHRF with three gamma functions enables the visualization of functional activation of the initial dip duration. In this paper, using MATLAB’s robustfit command, the *t*-values were determined. The activation in the functional maps were decided for *t*-value > *t*_crt_ and *p*-value < 0.05 or 0 for other *t*-values. The *t*_crt_ depends on the degrees of freedom of the data. In this study, the value 1.65 is utilized for *t*_crt_ (i.e., for 30 s trial, 275 data points (30 s × 9.19 Hz), 274 degrees of freedom). In order to show activation in the covered brain area, the resulting *t*-values were normalized within the 0~1 range and shown on a *t*-map as a functional activation map. All the images were constructed at 227 × 227 pixel size. Only ∆HbO data were used for further analysis since several prior research has demonstrated that ∆HbO is a more sensitive and accurate indicator [3,26,45].

**Figure 2:**
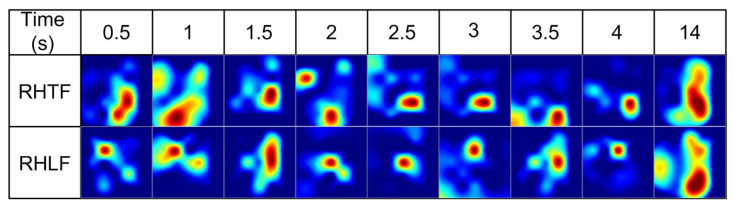
Functional *t*-maps at various time durations.

In this work, the functional *t*-maps for RHTF- and RHLF-tapping tasks were drawn for various initial dip durations (i.e., 0.5 s, 1 s, 1.5 s, 2 s, 2.5 s, 3 s, 3.5 s, and 4 s) and at 14 s for comparison with conventional delayed HR using the ∆HbO data. Figure 2 shows the functional *t*-maps drawn at different durations for RHTF- and RHLF-tapping tasks, respectively. Our hypothesis is that the functional *t*-maps acquired at the initial dip duration (i.e., 0.5 s, 1 s, 1.5 s, 2 s, 2.5 s, 3 s, 3.5 s, and 4 s) are more spatially precise than that of the functional *t*-map produced at 14 s and yield better classification accuracy for the tapping tasks acquired from the same brain area.

### 2.3. Isolated Convolutional Neural Network

CNN is the most widely used deep learning model in neural networks. The two components of a typical CNN model are feature extraction and classification. The input layer, convolution layer, pooling layer, fully connected layer, and classification layer are the five key layers of a CNN architecture. Through successively arranging trainable layers one after the other, CNN conducts feature extraction and classification. Convolutional and pooling layers are often included in the feature extraction section of a CNN, whereas fully connected and classification layers are typically included in the classification section. CNNs have been widely employed in many different fields whose input data are other than images, such as audio and video [46].

**Figure 3:**
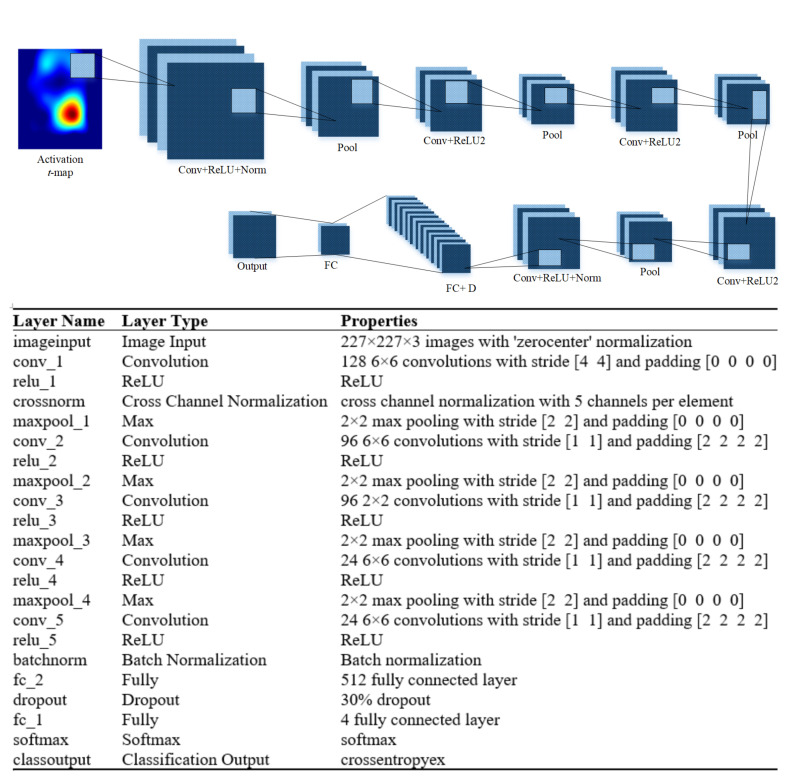
Proposed isolated CNN model to classify the functional *t*-maps of tapping tasks.

In this work, the four CNN models (16-layers, 19-layers, 22-layers, and 25-layers) are designed to classify the functional *t*-maps of RHTF- and RHTL-tapping tasks from the left motor cortex. The isolated CNN models acquire their input from RHTF and RHTL functional *t*-maps. CNN models with 16-layers, 19-layers, 22-layers, and 25-layers are designed by combining input, convolution, ReLU, normalization, maxpool, dropout, and fully connected layers. Figure 3 illustrates the 22-layered CNN model’s architect with a detailed description of each layer.

## 3. Results

**Figure 4:**
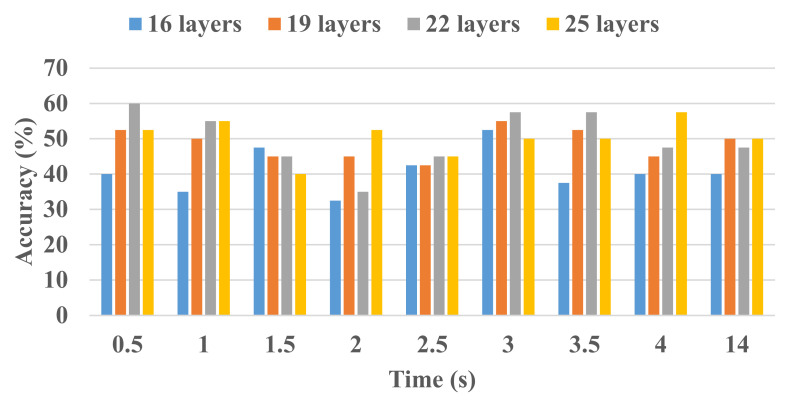
Classification accuracy for different layers’ (16-layers, 19-layers, 22-layers, and 25-layers) isolated CNN architecture at various time durations.

All the processing was conducted using MATLAB^TM^ 2021a. Firstly, the functional *t*-maps of RHTF- and RHLF-tapping tasks generated at various intervals for the initial dip duration (i.e., 0.5–4 s with a uniform interval of 0.5 s) and delayed HR at 14 s are individually fed (i.e., 11 subjects x 6 trials of RHTF/RHLF = 66 RHTF functional *t*-maps and 66 RHLF functional *t*-maps for each interval) to the isolated CNN designed using different layers (i.e., 16-layers, 19-layers, 22-layers, and 25-layers). For all models, 100 epochs, 0.01 initial learn rate, 0.9 momentum, and SGDM solver were used. In order to avoid the over-fitting of data, the data of each activity (i.e., RHTF and RHLF) were divided into a 70:30 ratio (70% data for training and 30% data for validation). Figure 4 shows the accuracy for different-layered isolated CNN architecture at various time durations. Isolated CNN with 22-layers yielded the best accuracy with less complexity. However, the classification accuracy is lower than acceptable (i.e., 70%). In most cases, the classification accuracy is also below the chance level (i.e., 50%).

**Figure 5:**
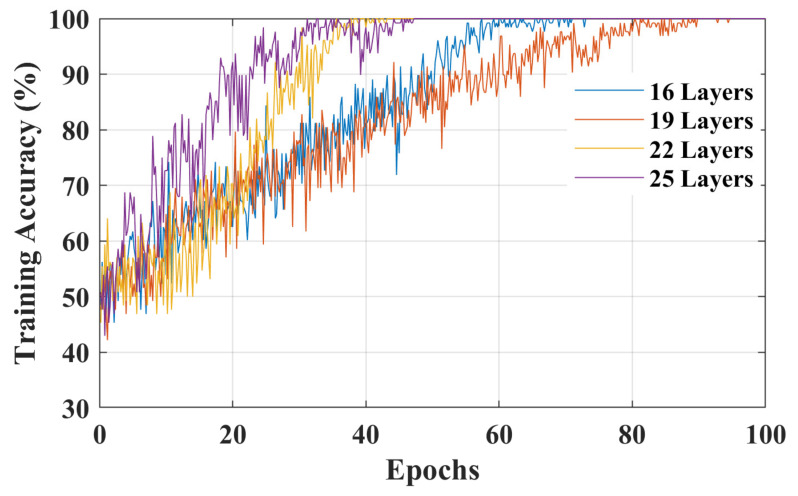
Classification accuracy for 16-layers, 19-layers, 22-layers, and 25-layers of isolated CNN architecture using the functional *t*-maps of initial dip (i.e., 0.5 to 4 s with a uniform interval of 0.5 s).

**Figure 6:**
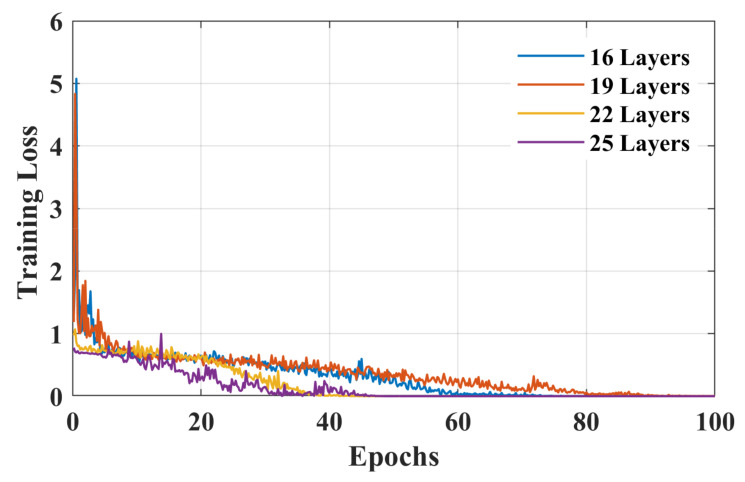
Loss curves for 16-layers, 19-layers, 22-layers, and 25-layers of isolated CNN architecture using the functional *t*-maps of initial dip (i.e., 0.5 to 4 s with a uniform interval of 0.5 s).

**Figure 7:**
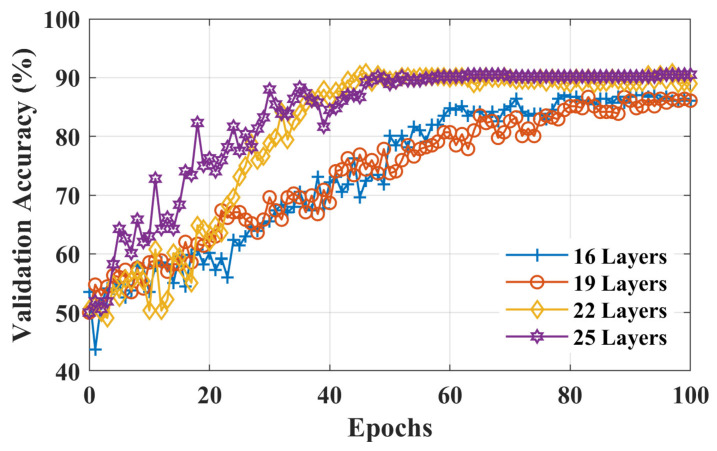
Validation accuracy for 16-layers, 19-layers, 22-layers, and 25-layers of isolated CNN architecture using the functional *t*-maps of initial dip (i.e., 0.5 to 4 s with a uniform interval of 0.5 s).

To increase the classification accuracy for initial dip duration, functional *t*-maps obtained at various intervals were combined to increase the size of the dataset (i.e., 11 subjects × 6 trials of RHTF/RHLF × 8 intervals = 528 RHTF function t-maps and 528 RHLF functional *t*-maps). Out of 528 images of each activity, 370 images were used to train the model, whereas the remaining unseen images were used for the validation of the model. The classification accuracy, loss curves, and validation accuracy of the training stage to distinguish RHTF- and RHLF-tapping tasks of the left motor cortex using the initial dip durations for different layers of isolated CNN are shown in Figure 5, Figure 6 and Figure 7 respectively. The validation accuracy of 85.8%, 86.1%, 89.2%, and 89.9% were obtained for 16-layers, 19-layers, 22-layers, and 25-layers of isolated CNN models for the two classes (i.e., RHTF- and RHLF-tapping tasks).

The 25-layered isolated CNN architecture yielded a classification accuracy of 89.9%, but the complexity of CNN architecture also increased. In comparison to the 25-layered isolated CNN network, it can be noted from Figure 5, Figure 6 and Figure 7 that the isolated CNN architecture with 22-layers yielded an accuracy of 89.2% for the classification of RHTF- and RHLF-tapping tasks for the initial dip durations from the same small brain area (i.e., left motor cortex) with low complexity and fast convergence. Furthermore, true positive rate (TPR), false negative rate (FNR), positive predictive value (PPV), and false discovery rate (FDR) metrics were calculated using Equation (1) for the selection of network layers, as shown in Table 1.

(1) $$\left.\begin{array}{c} \mathrm{TPR}\;\left(\%\right)=\frac{\mathrm{True}\;\mathrm{positive}}{\mathrm{No}.\;\mathrm{of}\;\mathrm{real}\;\mathrm{positive}}\times 100\\ \mathrm{FNR}\;\left(\%\right)=\frac{\mathrm{False}\;\mathrm{negative}}{\mathrm{No}.\;\mathrm{of}\;\mathrm{real}\;\mathrm{positive}}\times 100\\ \mathrm{PPV}\;\left(\%\right)=\frac{\mathrm{True}\;\mathrm{positive}}{\mathrm{True}\;\mathrm{positive}+\mathrm{False}\;\mathrm{positive}}\times 100\\ \mathrm{FDR}\;\left(\%\right)=\frac{\mathrm{False}\;\mathrm{positive}}{\mathrm{True}\;\mathrm{positive}+\mathrm{False}\;\mathrm{positive}}\times 100 \end{array}\right\}$$

**Figure 8:**
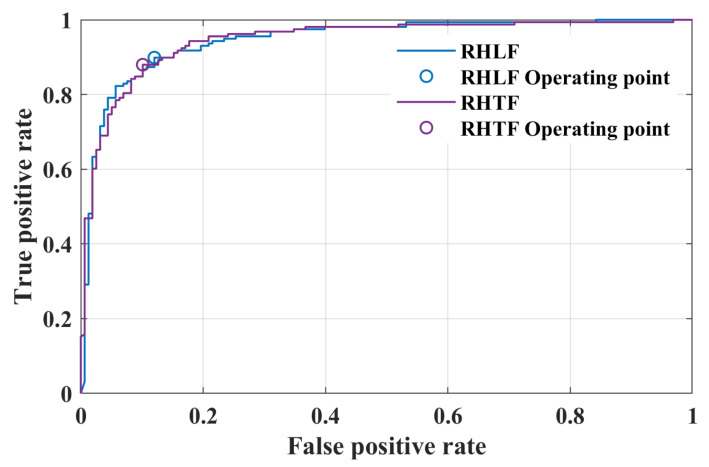
AUC for 22-layered isolated CNN architecture for validation stage using the functional *t*-maps of initial dip (i.e., 0.5 to 4 s with a uniform interval of 0.5 s).

Table 1 shows the confusion matrix of the validation stage for 16-layers, 19-layers, 22-layers, and 25-layers of isolated CNN models. It can be seen that the 22-layered isolated CNN architecture correctly classified 142 unseen images of RHTF and 140 unseen images of RHLF-tapping tasks, respectively. It has a TPR of 88.6% and 89.9% for RHLF and RHTF, respectively, whereas the 25-layered model has less TPR for RHTF than the 22-layered model and has a high TPR for the RHLF class. After carefully analyzing the results, it was noted that the 25-layered model has not significantly increased the model accuracy compared to the 22-layered model, but the complexity of the model was increased. Therefore, the area under the curve (AUC) of the 22-layered isolated CNN architecture (proposed model) is shown in Figure 8.

## 4. Discussion

The novelties of this work are two-fold: (i) the designing of an isolated CNN network for the fNIRS data and (ii) using the initial dip duration (0.5 to 4 s with a uniform interval of 0.5 s) functional *t*-maps to classify the finger-tapping tasks from the same small brain area (left motor cortex).

In a previous study by [43], the classification accuracy of 74.9% ± 6.4% was obtained for the same RHTF- and RHLF-tapping tasks using the time domain features of the initial dip duration. However, our hypothesis in this study was that the images created using the initial dip duration would give better classification accuracy for the activities obtained from the same small brain area. Furthermore, CNN provides the further advantage of automatic feature extraction by learning the image data and giving better accuracy. In this paper, the functional *t*-maps were drawn using the three gamma functions. The initial dip was included in the dHRF model with three gamma functions, adding an additional degree of freedom and improving the fNIRS signal’s estimate and prediction [26]. The obtained results supported the hypothesis by showing that the classification accuracy is increased to 89.2% for the classification of RHTF- and RHLF-tapping tasks by using the initial dip images (see Figure 5, Figure 6 and Figure 7). Our findings are in line with the previous literature showing that the initial dip duration reflecting the extraction of oxygen at the task initiation is spatially more specific than the delayed HR [9,22,43,44,47]. In comparison to initial dip images, the classification accuracy decreased using the delayed HR (images drawn at 14 s), which shows that it is difficult to classify the different tasks originating from the small brain area due to an increase in cerebral blood flow [9,22,48,49]. 

In recent years, deep learning has been successfully applied for pre-processing/augmentation, brain–computer interface (BCI) applications, brain response analysis, and diagnosis using the fNIRS signals [30]. However, all aforementioned studies focused on the conventional delayed HR (different temporal windows) obtained for various activities from the different brain areas [28,29,31,39,41,42]. In comparison, this study is focused on the initial dip duration (0.5 to 4 s with a uniform interval of 0.5 s) and classification of finger-tapping tasks (thumb and little) from the same small brain area. Furthermore, in this study, different layers of isolated CNN architecture (i.e., 16-layers, 19-layers, 22-layers, and 25-layers) were tested for fNIRS signals (thumb- and little finger-tapping) obtained from the left motor cortex. Initially, all networks yielded low accuracy when trained with images obtained at various time durations (i.e., 0.5–4 s with an interval of 0.5 s and 14 s). The reason might be the small size of images (i.e., 66 images for each task) and fewer details for the initial dip duration. Several previous studies suggest that the initial dip duration peaks at 2 s and are complete at 4 s [9,50,51,52]. Therefore, the size of the dataset was increased by combining all images obtained at various duration from 0.5 to 4 s (i.e., 528 images for each task). The results show that the isolated CNN trained well using the images of different durations, enabling the proposed model to yield an accuracy of 89.2% for unseen images (see Table 1). Furthermore, Figure 8 also shows that both tasks were classified with almost similar classification rates. Our result suggests that the brain response due to different tasks originating in the same small brain area can be well classified using the initial dip images.

Finally, this study demonstrates that the images obtained for the initial dip duration are spatially more specific for the activities originating from the same small brain area. The initial dip duration image depicts the fast oxygen extraction at the start of the task initiation. These results will be helpful in the future for diagnosis or critical analysis of patients with brain injuries. The limitation of this study is that only functional t-maps and ∆HbO were used for analysis. Therefore, other markers for generating functional images should be investigated in the future. Furthermore, the analysis using ∆HbR, change in cerebral oxygen exchange (∆COE), and change in cerebral blood volume (∆CBV) should be incorporated to enhance the classification accuracy for more accurate cortical analysis.

## 5. Conclusions

In this study, initial dip-based functional images and isolated CNNs were used to classify the right-hand finger-tapping tasks (thumb and little) of the same brain area (left motor cortex) using fNIRS signals. Various layers (i.e., 16-layers, 19-layers, 22-layers, and 25-layers) of isolated CNN were designed. All isolated CNN models were trained on the functional *t*-maps of the right hand’s thumb- and little finger-tapping tasks generated using three gamma functions-based designed HR; the 22-layered CNN yielded the highest accuracy. The results demonstrated that the two tapping tasks of the same brain area are quite distinguishable, with an accuracy of 89.2% using the initial dip-based functional *t*-maps drawn for 0.5 to 4 s with a uniform interval of 0.5 s. This study shows that the brain response due to different tasks originating in the same small brain area can be well classified using the initial dip images and can be useful in the future for fNIRS-based diagnosis or cortical analysis.

## Figures and Tables

**Table 1:** Performance of various network layers (i.e., 16-layers, 19-layers, 22-layers, and 25-layers) to classify functional *t*-maps of initial dip (i.e., 0.5 to 4 s with a uniform interval of 0.5 s).

Network Layers	Class	Classified as		TPR (%)	FNR (%)	PPV (%)	FDR (%)	Accuracy (%)
		RHLF	RHTF					
16	RHLF	136	22	86.1	13.9	85.5	14.5	85.8
	RHTF	23	135	85.4	14.6	86.0	14.0	
19	RHLF	134	24	84.8	15.2	87.0	13.0	86.1
	RHTF	20	138	87.3	12.7	85.2	14.8	
22	RHLF	140	18	88.6	11.4	89.7	10.3	89.2
	RHTF	16	142	89.9	10.1	88.8	11.3	
25	RHLF	144	14	91.1	8.9	88.9	11.1	89.9
	RHTF	18	140	88.6	11.4	90.9	9.1	

## Data Availability

The data used to support the findings of this study are included in the article.
